# Ratio of mitochondrial to nuclear DNA affects contamination estimates in ancient DNA analysis

**DOI:** 10.1038/s41598-018-32083-0

**Published:** 2018-09-19

**Authors:** Anja Furtwängler, Ella Reiter, Gunnar U. Neumann, Inga Siebke, Noah Steuri, Albert Hafner, Sandra Lösch, Nils Anthes, Verena J. Schuenemann, Johannes Krause

**Affiliations:** 1 Institute for Archaeological Sciences, Archaeo- and Palaeogenetics, University of Tübingen, Tübingen, Germany; 2 Department of Physical Anthropology, Institute of Forensic Medicine, University of Bern, Bern, Switzerland; 3 Institute of Archaeological Sciences and Oeschger Centre for Climate Change Research, University of Bern, Bern, Switzerland; 4 Institute of Ecology and Evolution, Animal Evolutionary Ecology group University of Tübingen, Tübingen, Germany; 5 Institute of Evolutionary Medicine, University of Zurich, Zurich, Switzerland; 6 Senckenberg Centre for Human Evolution and Palaeoenvironment, University of Tübingen, Tübingen, Germany; 7 Max Planck Institute for the Science of Human History, Jena, Germany

## Abstract

In the last decade, ancient DNA research has grown rapidly and started to overcome several of its earlier limitations through Next-Generation-Sequencing (NGS). Among other advances, NGS allows direct estimation of sample contamination from modern DNA sources. First NGS-based approaches of estimating contamination measured heterozygosity. These measurements, however, could only be performed on haploid genomic regions, i.e. the mitochondrial genome or male X chromosomes, but provided no measures of contamination in the nuclear genome of females with their two X chromosomes. Instead, female nuclear contamination is routinely extrapolated from mitochondrial contamination estimates, but it remains unclear if this extrapolation is reliable and to what degree variation in mitochondrial to nuclear DNA ratios affects this extrapolation. We therefore analyzed ancient DNA from 317 samples of different skeletal elements from multiple sites, spanning a temporal range from 7,000 BP to 386 AD. We found that the mitochondrial to nuclear DNA (mt/nc) ratio negatively correlates with an increase in endogenous DNA content and strongly influenced mitochondrial and nuclear contamination estimates in males. The ratio of mt to nc contamination estimates remained stable for overall mt/nc ratios below 200, as found particularly often in petrous bones but less in other skeletal elements and became more variable above that ratio.

## Introduction

The emergence of Next-Generation-Sequencing (NGS) technologies has substantially advanced the field of ancient DNA (aDNA) research^1^. Besides its high throughput, NGS gave rise to analyses of ancient DNA-specific DNA damage to reveal patterns that authenticate ancient DNA. *Post-mortem* DNA continuously accumulates characteristic modifications, in particular deamination at the DNA fragment ends^2–4^. Deamination frequency thus increases over time and can therefore reveal the ancient origin of an aDNA sample^3^. As a second step required for authenticating ancient human DNA, NGS data allow estimating contamination levels of human DNA directly^1^. Earlier estimates of contamination levels in NGS data from early modern humans were based on so-called diagnostic positions on the mitochondrial DNA (mtDNA), i.e. nucleotide positions that differ between the sample and a comparative dataset of complete modern human mtDNA sequences from world-wide populations^5^. Reads exhibiting a different allele at these positions than the majority of reads likely constitute contamination. Today, Bayesian approaches allow even more precise estimates by identifying the proportion of sequencing reads that were considered to be authentic aDNA from the studied individual^6^.

While quantifications of contamination levels in the haploid and maternally inherited mtDNA are achieved by measuring levels of heterozygosity^1^, contamination estimates for the diploid nuclear genome can only be obtained for the haploid sex chromosomes in male individuals, especially for the larger X chromosome^7^. For female individuals, most current studies therefore restrict analyses to nuclear DNA sequences with typical aDNA damage patterns and thus sequences of trusted ancient origin, often reducing datasets by an order of magnitude^8^. Alternatively, female mtDNA contamination estimates are extrapolated to the nuclear level and only female samples with low mtDNA contamination estimates are used for population genetic analysis on the nuclear genome. Whether this extrapolation is reliable, however, remains untested, in particular given that mitochondrial to nuclear DNA ratios can substantially vary between and even within bone samples^9^, potentially affecting the extrapolation of mtDNA to nuclear contamination levels.

The reliability of this extrapolation may also depend on the chosen skeletal element given differences in endogenous DNA content and relative amounts of mitochondrial and nuclear DNA. Recent work has identified the petrous part of the temporal bone as a particularly rich source of endogenous DNA and thus an ideal candidate for aDNA studies^10,11^. Endogenous DNA portions in this skeletal element exceed that in other parts of the skeleton by up to a factor of 400^12^. This reduces the cost of shotgun sequencing for ancient human genomes to sufficient coverage^10^ and allows obtaining sequences from geographic regions with typically poor DNA preservation, such as the Near East, Remote Oceania and Africa^13–17^. The exceptionally good DNA preservation in petrous bones has been linked to the high bone density of the bony labyrinth^10^, which reaches adult size during gestation^18^ and shows reduced bone remodeling compared to the surrounding tissue^19^. These conditions seem to be ideal for DNA preservation. Initial studies suggest low mt/nc ratios for petrous bones^20^. We hypothesize that these low ratios allow reliable estimates of contamination with human DNA using mtDNA, especially for female individuals.

This is the first study to explore systematic differences in mt/nc ratios between skeletal elements and classically used negative controls, and the relationship between mt/nc ratios and contamination estimates. We compare mt/nc ratios and overall DNA preservation in petrous bones, teeth and other skeletal elements in newly produced and publically available aDNA datasets. Our data show that DNA contamination estimates in males strikingly vary with mt/nc ratios, and argue that the typically low mt/nc ratios in petrous bones make them ideal for making reliable contamination estimates using mtDNA.

## Results

### Differences in the mt/nc ratio between sites

**Figure 1:**
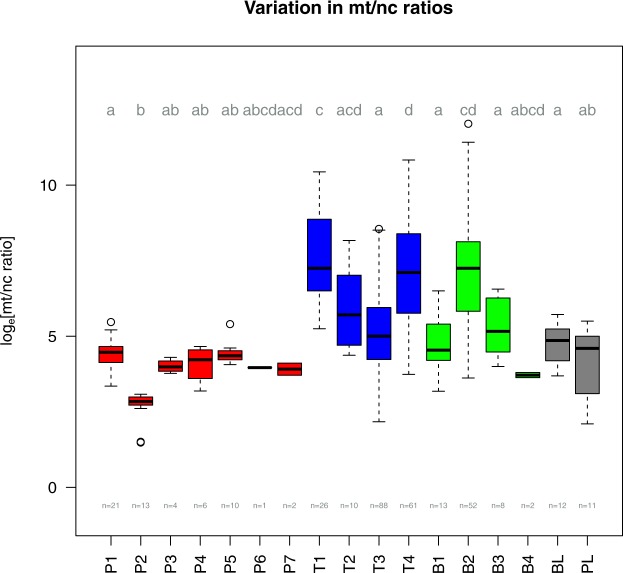
Variation in mt/nc ratios between sample sources. Mitochondrial to nuclear DNA ratios (log-transformed) are shown for petrous bones (red), teeth (blue), other bones (green), and controls (grey). Box plots show the raw data median (thick line), interquartile range IQR (box), data within 1.5*IQR (flags), and extreme values (dots). Different lower case letters indicate pairwise differences revealed by Tukey-HSD post-hoc tests.

For each of the three tested skeletal elements we investigated samples from different sites (Table 1), labelled P1 through P7 for petrous bones, T1 through T4 for teeth, and B1 through B4 for other bones. The subsample pairs T3/B1, T4/B2 and P1/T1 originate from the same studies, respectively. Given that any systematic differences between sample origins in, for example, general preservation, laboratory protocols, soil type, climatic conditions and taphonomic alterations can introduce between-sample variation, we first assessed the degree of origin-related variation in mt/nc ratios within each skeletal element (Fig. 1). Indeed, we detected significant variation between sample origins in all skeletal elements (Petrous bones ANOVA F_6,50_ = 20.15, p < 0.001; Teeth ANOVA F_3,181_ = 25.25, p < 0.001; Bones ANOVA F_3,71_ = 9.26, p < 0.001), with at least one sample differing from all remaining ones in each skeletal element (Tukey-HSD tests, Fig. 1). In all the following statistical models comparing differences in mt/nc ratios between tissues, we therefore routinely – and conservatively – accounted for between-origin variation while maintaining the paired nature of samples from the same source by integrating study ID and site ID as random factors.

**Table 1:** Samples from different studies used in this analysis.

Site	Dating	Samples			Reference
		Petrous bones	Teeth	Diverse bones	
Oberbipp, Switzerland	approx. 5500 BP	P1	T1		This study
Spreitenbach, Switzerland	approx. 4500 BP		T2		This study
Europe and Central Asia	3400 BP–600 AD		T3	B1	^38^
Abusir el-Meleq, Egypt	1300 BP–386 AD		T4	B2	^39^
Hungary	5060–1830 BP	P2			^10^
Atapuerca, Spain	5500–3500 BP			B3	^40^
Zagros, Iran	10000–7000 BP	P3		B4	^13^
Zagros, Iran	10000–9700 BP				^14^
Muttenz, Switzerland	Neolithic	P4			This study
Wartau, Switzerland	Neolithic	P5			This study
Seengen, Switzerland	Neolithic	P6			This study
Bad Zurzach, Switzerland	Neolithic/Bronze Age	P7			This study

### Differences in the mt/nc ratio between skeletal elements

To assess the mt/nc ratio of potential sources for contamination of archaeological samples, we also measured the mt/nc ratio of human contamination originating from plant extracts^21^ and laboratory negative controls. If mt/nc ratios of modern human contamination exceeded those of the ancient human DNA, the relative contamination impact would be stronger on the mtDNA than on the nDNA, and *vice versa*. We found no significant difference in the log_e_[mt/nc ratio] between both controls (ANOVA F_1,21_ = 2.33, p = 0.14), therefore combining them for all further analyses.

**Figure 2:**
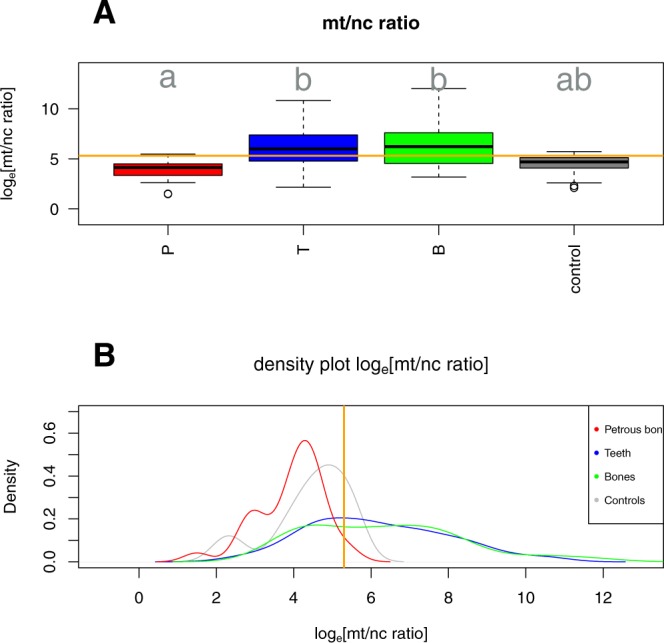
Variation in the mt/nc ratios between skeletal elements. (**A**) Box plots of the mitochondrial to nuclear DNA ratio (log-transformed) of human DNA in petrous bones (P), teeth (T), other bones (B) and controls. Panel (**B**) shows density plots of the same data across elements. Orange lines indicate the suggested threshold of the mitochondrial to nuclear ratio of 200 (log_e_(200) = 5.3).

The mt/nc ratios showed significant variation (Fig. 2) between the three skeletal elements (petrous bones, teeth and bones) and controls (Chi² = 9.54, df = 3, p = 0.023). Petrous bones had significantly lower mt/nc ratios than teeth and showed a trend towards lower mt/nc ratios than other bone samples, while teeth and other bones showed similarly high ratios (Tukey HSD post hoc tests as indicated in Fig. 2). The mt/nc ratios of the combined control group were intermediate between the ratios derived from teeth and bones and those derived from petrous bones. In addition to these differences in mean mt/nc ratios, skeletal elements also showed striking differences in mt/nc ratio variation (Levene’s test F_3,336_ = 15.01, p < 0.001). Variation was again similar between teeth and other bones (Tukey HSD post hoc P = 0.75) but significantly larger in these two than in petrous bones (both P < 0.0001).

### Factors influencing the mt/nc ratio

**Figure 3:**
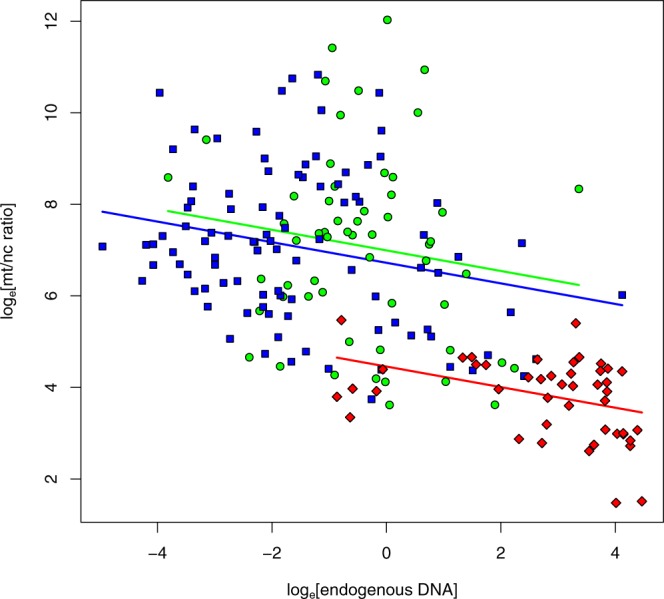
Relationship between the mt/nc ratio and the percentage of endogenous DNA in petrous bones (red), teeth (blue) and other bones (green). Despite overall differences in average mt/nc ratios between skeletal elements and subsamples, these ratios declined consistently when samples contained more endogens DNA.

To assess the degree to which mt/nc ratios vary with sample characteristics within sample origins, we tested relationships with endogenous DNA content, age, and mapping stringency. Despite much between-sample variation, mt/nc ratios almost consistently decreased with increasing endogenous DNA content across skeletal elements and sampling origins (Fig. 3, mixed model endogenous DNA-effect Chi² = 6.57, df = 1, P = 0.010, overall regression slope = −0.224 ± 0.082, mean ± SE).

In contrast, we detected no significant relationship between radio carbon dates of the P2, P3, T3 and B2 samples and their log_e_[mt/nc ratio] (mixed model sample age-effect Chi² = 0.63, df = 1, P = 0.426). Changes in the mapping stringency in terms of mismatches allowed per read (BWA parameter *–n*, with tested values 0.001, 0.01, 0.1, 0.2 and 0.8 corresponding to 5, 3.7, 2, 1.7 and 0 mismatches for the average fragment size of T1 and T2 of 55 bp) showed some fluctuation in the mt/nc ratio in T1 and T2 teeth, but no directional effect on mean mt/nc ratios (linear regression F_1,125_ = 2.049, P = 0.155, R² = 0.016).

### Effect of changes in mt/nc ratio on contamination estimates

**Figure 4:**
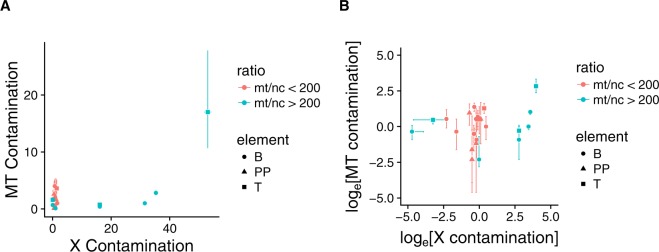
Relationship between male mitochondrial and X-chromosome contamination rates in un-transformed (**A**) and log_e_ transformed (**B**) data. Different colours indicate samples with low (<log_e_(200) = 5.3 red) and high (>log_e_(200) = 5.3 blue) mt/nc ratios.

To evaluate whether mitochondrial and nuclear contamination estimates vary with sample mt/nc ratios, we analysed male individuals where sufficient NGS coverage allowed reliable estimates of mtDNA and nuclear contamination levels. Mitochondrial and X-chromosomal contamination rates were by far most strongly biased towards mt-contamination in samples with high mt/nc ratios (Fig. 4). In contrast, where mt/nc ratios were small, nuclear contamination estimates were close to mtDNA contamination rates with an average ratio of 1.47 ± 0.44 SE. In addition, contamination ratios remained stable across log_e_[mt/nc ratio] between 0 and log_e_(200) = 5.3 (mixed model log_e_[mt/nc ratio]-effect Chi^2^ = 0.80, df = 1, p = 0.37), but strikingly increased with log_e_[mt/nc ratio] at values exceeding log_e_(200) = 5.3 (Chi² = 5.95, df = 1, p = 0.015). This stability is reflected in the fact that samples with a ratio below log_e_(200) = 5.3 are located on the bisecting line when plotting MT contamination against X contamination (Fig. 4).

## Discussion

In this work, we show that variation in the mt/nc ratio of ancient DNA samples strongly influences the estimates of contamination by human DNA, which is one of the most critical steps when analysinig ancient human DNA. Besides the characteristic age related misincorporation^2^, estimates of contamination rates are used for authentication of ancient human DNA. Currently available methods for contamination estimates of the mtDNA and nDNA levels measure heterozygosity in haploid regions of the mtDNA, or the X chromosome in males^6,22,23^. However, at present it is not possible to estimate contamination levels in the nDNA of female individuals. Recent studies of ancient human nDNA either restrict their analysis to male individuals with known nDNA contamination levels or extrapolate contamination levels of mtDNA to nDNA in the case of females.

Our results show that contamination levels extrapolated from the mtDNA to the nDNA might systematically underestimate the actual level of nDNA contamination. This appears particularly likely for teeth and other bones with a high mt/nc ratio, where such extrapolations (as often done for female specimens) may be highly misleading. For example, three “other bone” samples in the current study exhibited less than 3% contamination on the mtDNA, contrasting to nDNA-contamination between 16 and 30% measured on the X-chromosomes. All three samples were characterized by high mt/nc ratios spanning 870 to >56.000. For these samples, it is not possible to determine from the distribution of reads on the X and Y chromosome with certainty if they are males with a contaminated X chromosome or if they are females with male contamination. Either way, the contamination on the nuclear level is too high for further population genetic analysis and even in absence of Y chromosomal reads as an indicator of contamination, the extrapolation from the mtDNA contamination level could still underestimate the actual nuclear contamination level in case of high mt/nc ratios. Therefore, we advise to take the mt/nc ratio into consideration if only the mtDNA contamination estimate is used in order to choose female samples to be included in population genetic analysis. An effect of mt/nc ratio on the contamination estimates of mtDNA and nDNA as described above was already suggested in the context of analysing Neandertal nuclear and mtDNA^9,24^ and receives further support by our statistical analysis.

In contrast to samples with a high mt/nc ratio, samples with a ratio below 200 (log_e_[200] = 5.3) seem to provide a rather reliable estimate of contamination on the mitochondrial level (Fig. 4). We included two types of possible contamination in our study: modern human DNA contamination in archaeological samples measured from aDNA plant extracts from 6,000 year old barley^21^ and contamination introduced during lab work measured from the negative controls carried along in the entire laboratory workflow. The mt/nc ratios of these potential contamination sources were intermediate to those detected in the three tested skeletal elements (Fig. 2). Therefore, these types of contamination contribute more to the contamination level of mtDNA than nDNA in samples showing lower ratios than these controls. Our systematic comparison of a large number of teeth, diverse bones and petrous bones indicates that the petrous bones display relatively low mt/nc ratios and hence have a high chance to show even lower contamination rates on the nDNA than extrapolated from their mtDNA. In addition, we found that the mt/nc ratio in petrous bones not only tended to be smaller compared to teeth and other bones but also exhibited significantly less variation (Fig. 2). The lower mt/nc ratios in petrous bones increase the chance to obtain reliable extrapolations of nDNA contamination rates from estimated mtDNA contamination. Furthermore, the extraordinarily good preservation of endogenous DNA in petrous bones makes it possible to overcome the limited preservation of aDNA in most environments^12^. Hansen and colleagues^20^ show that teeth can have even higher contents of endogenous DNA than petrous bones. In our dataset, however, the majority of petrous bones displayed clearly higher endogenous DNA contents than teeth, leading us to maintain petrous bones as the prime source for highly concentrated and little contaminated endogenous DNA.

From these influences of the mt/nc ratio on the contamination estimates, we conclude that the mt/nc ratio should be considered if mtDNA contamination estimates are used to select female samples for nDNA studies. Reasons for the observed variation in this ratio have already been subject of different studies and no clear reason for the elevation of the mt/nc ratio in some aDNA samples compared to modern DNA has been identified. One possibility could be a bias of the mt/nc ratio originating from bacteria, since mtDNA contains features of a bacterial chromosome and bacterial reads from the environmental background might map better to the mtDNA elevating the mt/nc ratio. However, mapping with different stringencies (different numbers of allowed mismatches per read, BWA parameter *-n*) did not reveal a particular trend. Allowing more mismatches (*-n* 0.001, *-n* 0.01 and *-n* 0.1) resulted in similar values in the ratio than more stringent mapping (*-n* 0.2 and *-n* 0.8). We could therefore not detect any effect of the bacterial background in aDNA extracts on the correlation between high mt/nc ratios and lower percentages of endogenous DNA.

It has been suggested that the higher ratios in aDNA might originate from better protection of DNA in the mitochondrion due to its double membrane^25^. In addition, Schwarz and colleagues^25^ and Allentoft and colleagues^26^ conclude from their observation of longer mtDNA fragments compared to nDNA that mtDNA decays more slowly than nDNA. This would result in increasing ratios through time. No such trend is detectable in our data for petrous bones (P2, P3) and teeth (T3), and only a weak trend in other bones (B2). Therefore, we found no indication that different decay rates cause the observed variation in the mt/nc ratio.

Another reason for differences in mt/nc ratios could be higher metabolic activity in particular skeletal elements, as suggested by Higgins and colleagues^27^ in their study of different parts of teeth. Their observation indicates that higher mt/nc ratios in dentine may originate from its higher metabolic activity as compared to enamel and cementum. This assumption, of different metabolic activity resulting in different mt/nc ratios could also be applied to other skeletal elements, the petrous bone in particular. In the cortical part of the petrous bone, bone remodelling is suppressed compared to surrounding tissue by a specific signalling-pathway, and the number of metabolic active osteoclasts and osteoblasts is reduced^28^. Furthermore, the more compact bone regions contain a higher number of osteocytes, which have lower numbers of mitochondria^29^. The number of mitochondria in osteocytes decreases from periosteal and endosteal surfaces towards the inner and denser bone parts^30^. Consequently, sampling the hardest part of the petrous bone results in low mt/nc ratios due low mt/nc ratio in this area *ante-mortem*. Within the densest parts of the cortical bone displaying low mt/nc ratios, the endogenous DNA is also better protected against the environmental background of bacteria, fungi and other microbes resulting in high percentages of endogenous DNA.

In conclusion, we showed that the mt/nc ratio is an important value in aDNA authentication. It strongly influences the accuracy of extrapolating nDNA contamination levels from mtDNA contamination estimates. This approach should be used with particular caution if mt/nc ratios exceed 200. Lower mt/nc ratios are often associated with high percentages of endogenous DNA, as typically found in the densest parts of the petrous bones. In this case, low mt/nc ratios are most likely derived from a low mtDNA concentration in this bone region *ante-mortem*, which is caused by a low metabolic activity.

Temporal bones, including the petrous bones have already proven to be valuable in physical anthropology. The compact structure of the petrous bone is ideal for preservation in archaeological contexts, therefore, it can be used to estimate the minimal number of individuals^31^. While age can only be roughly estimated depending on developmental stages^32^. In addition, the discovery of the higher percentage of endogenous DNA in petrous bone compared to other skeletal elements^10,11^ constitutes a substantial improvement for the field of aDNA. Our study confirms those findings and shows that mitochondrial to nuclear ratios provide a further argument to extract ancient DNA from petrous bones providing reliable human contamination assessment from both sexes.

## Material and Methods

### Dataset

We combined shotgun data from 76 new ancient DNA samples from the late Neolithic dolmen burial site of Oberbipp in Switzerland^33,34^, the late Neolithic multiple burial of Muttenz^35^, the final Neolithic sites in Switzerland Spreitenbach^36^, Seengen^35^, Zurzach^35^ and cave finds from Wartau^37^ (detailed description of archaeological sites and laboratory workflow SI section 1 and 2) with published datasets (Table 1). In total, DNA from 317 ancient individuals and 23 controls was analysed for its mtDNA to nDNA (mt/nc) ratio. For each of the three investigated skeletal elements, samples originated from three sources. Different skeletal elements do not originate from the same individuals. Samples for petrous bones (n = 57) included 40 newly processed samples from the previous mentioned Swiss burials (P1, P4, P5, P6, P7), 13 from Gamba and colleagues^10^ (P2) and four from Broushaki and colleagues^13^ and Gallego-Llorente and colleagues^14^ (P3). Teeth samples (n = 185) included 36 from the Swiss burials (T1, T2), 88 from Allentoft and colleagues^38^ (T3) and 61 from Schuenemann and colleagues^39^ (T4). P1 and T1 from this study originate from the same multiple burial in Oberbipp, Switzerland but due to the comingled nature of the remains an assignment of the teeth to the petrous bones is not possible. Furthermore, the endogenous DNA in the teeth is too low to allow kinship analysis to identify identical individuals. Therefore, it is possible that teeth and petrous bones are from the same individuals. Finally, diverse bone samples from compact bone parts (n = 75) included 13 from Allentoft and colleagues^38^ (B1), 52 from Schuenemann and colleagues^39^ (B2) and eight from Günther and colleagues^40^ (B3) and two from Broushaki and colleagues^13^ (B3). Fastq files were downloaded from the European Nucleotide Archive. Radio carbon dates were used from Gamba and colleagues^10^, Broushaki and colleagues^13^, Gallego-Llorente and colleagues^14^ and Schuenemann and colleagues^39^.

### Bioinformatic processing

All data were processed with the EAGER pipeline^41^. If necessary, adapters were removed and paired-end data was merged using Clip&Merge^41^. Mapping was performed with BWA with the mismatch parameter set to 0.01 and a seed length of 1,000. If necessary, PCR duplicates were removed using DeDup^41^. Mitochondrial to nuclear ratios were calculated by dividing the mean coverage of the mitochondrial genome by the mean coverage of the nuclear genome to take into account the different length of nuclear and mitochondrial genome. Samples with no reads on the mitochondrial chromosome resulting in a ratio of zero as found in some negative controls, plant extracts, and extremely bad preserved teeth samples were excluded.

Reads mapping to the mitochondrial genome were extracted from the BAM files and the mitochondrial genome was reconstructed using the software *schmutzi*^22^. Mitochondrial contamination was then estimated with a Bayesian approach as described in Fu and colleagues^6^. Sex determination was performed after Skoglund and colleagues^42^. X chromosomal contamination in males with more than 0.5-fold coverage on the X chromosome was estimated using ANGSD^23^.

### Statistical analysis

Statistical analysis was performed in R version 3.4.3 (R Core Team 2017). We first used one-way ANOVA to assess differences in log_e_[mt/nc ratio] (i.e., the raw mt/nc-ratios log_e_-transformed to approach normality) between the sub-samples within each of the three different skeletal elements and controls, followed by Tukey HSD *post hoc* tests. Second, to assess overall differences in log_e_[mt/nc ratio] between skeletal elements and controls, we performed linear mixed models as implemented in the lme4-package for R^43^. Given substantial variation in log_e_[mt/nc ratio] between different sample origins and studies detected above, this model contained the source study as a random slope factor, allowing between-study variation in the main effect while maintaining the paired nature of samples from the same source. Pairwise Tukey HSD *post hoc* tests were calculated from this model using the lsmeans package for R^44^. Differences in the variance of the log_e_[mt/nc ratio] between skeletal elements and control were tested using Levene’s test, followed by Tukey HSD *post hoc* tests.

To assess how log_e_[mt/nc ratio] varies with DNA-content (log-transformed to approach normality) we constructed a linear mixed model as above with log_e_[mt/nc ratio] as the response variable, log_e_[endogenous DNA] as the predictor, and subsample as random slope factor allowing differential regression slopes between subsamples. In addition, we added skeletal element and its interaction with log_e_[endogenous DNA] as fixed factor to assess slope consistency between skeletal elements. Given that this interaction was clearly insignificant (Chi² = 0.18, df = 2, P = 0.91) we removed it from the final reported model. Information about endogenous DNA content was only available for all Swiss and Egyptian samples. An identical model structure was used to assess the relationship between sample age and log_e_[mt/nc ratio], now with sample age as the main predictor.

Differences in log_e_[mt/nc ratio] between different mapping stringencies were assessed using linear regression. Information about log_e_[mt/nc ratio] at different mapping stringencies was obtained for the teeth of the Swiss burials sites Oberbipp and Spreitenbach (T1) since they have low endogenous content and a high portion of bacterial background.

Finally, to assess how the ratio of mitochondrial over X-chromosomal contamination varies with log_e_[mt/nc ratio] we constructed linear mixed models as above, but separate for samples with low (<5.3) and high (>5.3) log_e_[mt/nc ratio]. Contamination ratio served as the response variable, log_e_[mt/nc ratio] as the predictor, and skeletal element as a random slope factor allowing differential responses to log_e_[mt/nc ratio].

## Electronic supplementary material

Supplementary Information

Supplementary Dataset
