# Supplementary material for: Ratio of mitochondrial to nuclear DNA affects contamination estimates in ancient DNA analysis
Source: Sci Rep. 2018 Sep 19;8:14075. doi: 10.1038/s41598-018-32083-0 (PMC6145933; doi:10.1038/s41598-018-32083-0)
Supplement: Supplementary file 1 — Supplementary Information [file 41598_2018_32083_MOESM1_ESM.docx]

**Ratio of mitochondrial to nuclear DNA affects contamination estimates in ancient DNA analysis**

Anja Furtwängler^1*^, Ella Reiter^1^, Gunnar U. Neumann^1^, Inga Siebke^2^, Noah Steuri^3^, Albert Hafner^3^, Sandra Lösch^2^, Nils Anthes^4^, Verena J. Schuenemann^1,5,6^, Johannes Krause^1,6,7*^

^1^Institute for Archaeological Sciences, Archaeo- and Palaeogenetics, University of Tübingen, Germany

^2^Department of Physical Anthropology, Institute of Forensic Medicine, University of Bern, Switzerland

^3^Institute of Archaeological Sciences and Oeschger Centre for Climate Change Research, University of Bern, Switzerland

^4^Institute of Ecology and Evolution, Animal Evolutionary Ecology group University of Tübingen, Germany.

^5^Institute of Evolutionary Medicine, University of Zurich, Switzerland

^6^Senckenberg Centre for Human Evolution and Palaeoenvironment, University of Tübingen, Germany

^7^Max Planck Institute for the Science of Human History, Jena, Germany



* Corresponding author

E-mail: krause@shh.mpg.de, anja.furtwaengler@uni-tuebingen.de

**Supplementary Section 1 Archeological sites**

**Oberbipp, Canton Bern, Switzerland**

Ramstein et al. 2014, Siebke et al. 2017

The Dolmen burial from Oberbipp was discovered and excavated in 2012. First ^14^C results date to 5500 BP. The dolmen contains a minimal number of 40 individuals as determined from femora including males and females of all age classes. The burial is assigned to the Horgen Culture.

**Spreitenbach, Canton Aargau, Switzerland**

Doppler 2012

In 1997 the multiple burial of Spreitenbach, Canton Aargau in Switzerland was discovered and excavated. It contained five man, four women, two subadults and a new born. The burial is radio carbon dated to approximately 4500 BP and assigned to the Corded Ware complex.

**Muttenz, Canton Basel, Switzerland**

Bleuer et al. 2012

The multiple burial of Muttenz was discovered in 1969. It contained five individuals and was assigned to the Bell Beaker Complex.

**Wartau, Canton St. Gallen, Switzerland**

Stehrenberger 2016

The archaeological site is located in a natural cave. In the 1970s and 1980s, several inspections by archaeologist of the archaeological service of the Canton St. Gallen took place. The cave was accessible to the public at all time and in the 1970s some human remains were recovered illegally by private persons and transferred to the archaeological service in 2001. Radio carbon dates of bone fragments date to the Middle Neolithic period.

**Seengen, Canton Aargau, Switzerland**

Bleuer et al. 2012

The grave mound of Seengen was excavated in 1993. The burial contained two individuals, one of them consisted only of burned remains. The grave mound is archaeological dated to the Bronze Age.

**Bad Zurzach, Canton Aargau, Switzerland**

Bleuer et al. 2012, Gutzwiller 1994

The double burial of Bad Zurzach was excavated in 1984. It was assigned to the Bronze Age. Both individuals were buried in contracted position.

**Supplementary Section 2 Laboratory workflow**

40 petrous bones (P1, P4 - P7) and 36 teeth (T1) from the Neolithic sites in Switzerland were shotgun sequenced. Between 30 mg and 50 mg of powder from coronal dentin for teeth and between 50 mg and 100 mg of bone powder for the petrous bones was used for extraction as described previously (**Dabney et al. 2013)**. Sequencing libraries were prepared after **Meyer and Kircher 2010**. To enable multiplexed sequencing, sample specific barcodes were added to each library by amplification with tailed primers (**Kircher et al. 2011**). To achieve a high copy number of each library, an additional amplification was performed with Herculase II in a final volume of 100 μl consisting of 1 x Herculase II reaction buffer, 0.25 mMdNTPs, 0.4 μM IS 5, 0.4 μM IS 6, 0.01 % Herculase II Fusion DNA Polymerase. The thermal profile started with 2 min at 95 °C followed by sample specific number of cycles of 10 sec 95 °C, 30 sec 65°C, 30 sec 72 °C. This was then followed by 4 min at 72 °C. All samples were pooled equimolar at 10 nM and sequenced on a HiSeq with paired or single ends and NextSeq with paired ends. The mtDNA enrichment for MA315 and MA318 was performed as follows. For the generation of baits a long range PCR was performed on modern human mtDNA as described in **Maricic et al. 2010**. The purified products were sonicated with COVARIS shearing (Covaris, Woburn USA) to generate fragments of ~ 350 bp. For the preparation of the baits this step was followed by a blunt end reaction with Quick Blunting™ Kit of New England Biolabs and followed by a MinElute purification. To these fragments a double-stranded adapter was ligated generated from modified sequences after **Fu et al. 2013** according to the approach for generation of double-stranded adapters described in **Meyer and Kircher 2010**. The two adapters were generated from APL 5 (CGTGGATGAGGAGCCGCAGTG) and adapter1 rev (CACTGCGGCT) for the first adapter, the second adapter consists of APL6 (ATAGGGATCGCACCAGCGTGT) and adapter2 rev (ACACGCTGGT). Adapter ligation was performed using Quick Ligase (New England Biolabs) followed by a MinElute purification with an elution volume of 20 μl . For the fill in of the 5'-overhangs of the adapters the reaction had a total volume of 40 µl with 1 x Isothermal buffer, 125 nM dNTP, 0,4 U/μl Bst polymerase 2.0 from (BioLabs) and was incubated for 20 min at 37 °C and then 20 min at 80 °C. This was followed by an amplification within 8 reaction with Herculase II in a final volume of 100 μl consisting of 1 x Herculase II reaction buffer, 0,25 mM dNTPs, 0,4 μM APL 5, 0,4 μM APL 6, 0,01 % Herculase II Fusion DNA Polymerase and 30 cycles. Single-stranded probes were generated as in **Fu et al. 2013** using the APL2 primer. Before hybridisation 2 µg of target DNA were combined with 250 µM blocking oligonucleotides and incubated at 5 min at 95°C and 5 min at 65°C followed by at least 10 min at 37°C. This reaction was combined with 250 ng single-stranded bait of each fragment. The final hybridisation contains further more 1x HI-RPM hybridization buffer (aCGH Hybridization Kit; Agilent) and 1x Agilent blocking agent (aCGH Hybridization Kit; Agilent). The reaction is incubated over two nights at 65°C. For each hybridised library pool 20 µl of MyOne M-270 streptavidin beads (Invitrogen) were washed twice with 1xBWT and then resuspended in 20 µl BWT. The hybridisation reaction is added to the beads and 160 µl BWT are added and the reaction is incubated at RT for 30 min. The beads were then washed with 200 µl BWT three times and then washed twice with 200 µl preheated (60°C) HWT and an incubation for 2 min. This was followed by washing with 200 µl BWT and 100 µl TET. Then 15 µl TET were added and the copy number was determined by qPCR (End volume of 20 μl: 1X DyNAmo Mastermix and 0,5 μM IS7 and IS8 and temperature profile of 10 min at 95 °C, then 40 cycles of 30 sec at 95 °C, 30 sec at 60 °C and 30 sec at 72 °C) followed by another amplification using Herculase II both directly on the beads. For MA315 and MA318 two more libraries with a half UDG-treatment (**Rohland et al. 2014**) were prepared and enriched for 1,240k nuclear SNPs as described in **Schuenemann et al. 2017** to achieve sufficient coverage on the X chromosome. The libraries MA307 till MA381 were paired-end sequenced on a HiSeq4000 (2x75 cycles). The libraries MA395 till MA482 and the libraries SA65 till SA74 were single-end sequenced on a HiSeq4000 for 75 cycles.

**References**

1. Ramstein, M., Schimmelpfennig, D., Lösch, S. Ein neolithischer Dolmen an der Steingasse in Oberbipp*. Archäologie Schweiz* **37(3)**, 4-15 (2014).

2. Siebke I., Furtwängler A., Hafner A., Krause J., Lösch S. An Interdisciplinary Project on the Neolithic Population of Modern Switzerland. 86th Annual Meeting of the American Association of Physical Anthropologists. *American Journal of Physical Anthropology*. p.358 (2017)

3. Bleuer E, Doppler T, Fetz H.. Gräber im näheren und weiteren Umfeld von Spreitenbach in *Spreitenbach-Moosweg (Aargau, Schweiz): Ein Kollektivgrab um 2500 v Chr* (ed. Doppler, T.) 233-266 (Basel 2012)

4. Doppler, T. *Spreitenbach-Moosweg: ein Kollektivgrab um 2500 v. Chr*. Archäologie Schweiz, **Vol. 51** (2012).

5. Stehrenberger T. Obere Höhle bei der "Procha Burg" *Höhlenpost* **148** (2016).

6. Gutzwiller P. Die vorrömische Besiedlung des Fleckens Zurzach. *Jahrbuch der Schweizerischen Gesellschaft für IJr- und Frühgeschichte* **77**:7-33 (1994).

7. Dabney, J., et al. Complete mitochondrial genome sequence of a Middle Pleistocene cave bear reconstructed from ultrashort DNA fragments. *Proceedings of the National Academy of Sciences.* **39**, 15758–15763 (2013).

8. Meyer M., Kircher M. Illumina Sequencing Library Preparation for Highly Multiplexed Target Capture and Sequencing. *Cold Spring Harbor Protocols*. **5448** (2010).

9. Kircher, M., Sawyer, S.Meyer, M. Double indexing overcomes inaccuracies in multiplex sequencing on the Illumina platform. *Nucleic Acids Research.* **1**, e3 (2011).

10. Maricic, T., Whitten, M., Pääbo, S. Multiplexed DNA sequence capture of mitochondrial genomes using PCR products. *PloS ONE* ***5*(11)**, e14004 (2010).

11. Fu, Q., et al. DNA analysis of an early modern human from Tianyuan Cave, China. *Proceedings of the National Academy of Sciences* **110(6)**, 2223-2227 (2013).

12. Rohland N., Harney E., Mallick S., Nordenfelt S., Reich D. Partial uracil-DNA-glycosylase treatment for screening of ancient DNA. *Philosophical Transactions of the Royal Society B: Biological Sciences*. **370**, 20130624 (2014).

13. Schuenemann V.J., Peltzer A., et al. Ancient Egyptian mummy genomes suggest an increase of Sub-Saharan African ancestry in post-Roman periods. *Nat Comms*. **8**, 15694 (2017)
